# Side-effects of pesticides on the generalist endoparasitoid *Palmistichus elaeisis* (Hymenoptera: Eulophidae)

**DOI:** 10.1038/s41598-017-10462-3

**Published:** 2017-08-30

**Authors:** Ricardo Alcántara-de la Cruz, José Cola Zanuncio, Mabio Chrisley Lacerda, Carlos Frederico Wilcken, Flávio Lemes Fernandes, Wagner de Souza Tavares, Marcus Alvarenga Soares, Carlos Sigueyuki Sediyama

**Affiliations:** 1 Departamento de Entomologia/BIOAGRO, Universidade Federal de Viçosa, Viçosa, 36570-900 Brazil; 2 Embrapa Arroz e Feijão, Empresa Brasileira de Pesquisa Agropecuária, Santo Antônio de Goiás, 75375-000 Brazil; 3 Departamento de Proteção Vegetal, Universidade Estadual Paulista “Júlio de Mesquita Filho”, 18610-307 Botucatu, Brazil; 4 Instituto de Ciências Agrárias, Universidade Federal de Viçosa, 38810-000 Rio Paranaíba, Brazil; 5 Departamento de Fitotecnia, Universidade Federal de Viçosa, Viçosa, 36570-900 Brazil; 6 Departamento de Agronomia, Universidade Federal dos Vales do Jequitinhonha e Mucuri, 391000-000 Diamantina, Brazil

## Abstract

New plant protection strategies focus on minimizing chemical pesticide use and increasing their compatibility with biological control agents. The objective was to evaluate the side-effects of glyphosate, diflubenzuron, malathion, tebuconazole and triflumuron (at 720, 45, 400, 150 and 20 g ai ha^−1^, respectively), pesticides authorized for soybean crops in Brazil, on the parasitoid *Palmistichus elaeisis* (Hymenoptera: Eulophidae) reared on *Anticarsia gemmatalis* (Lepidoptera: Noctuidae). The emergence and female numbers produced per *P. elaeisis* female were higher in *A. gemmatalis* pupae from caterpillars fed an artificial diet treated with glyphosate. However, emergence was lower than 50% when the caterpillars were fed on soybean leaves treated with glyphosate offered *ad libitum* (3–5 times). *Palmistichus elaeisis* died before parasiting *A. gemmatalis* pupae treated with malathion. Diflubenzuron reduced the *P. elaesis* sex ratio in the second generation. Tebuconazole and triflumuron did not cause side-effects on this parasitoid. A continuous exposure to glyphosate by the host may lead to side-effects on *P. elaeisis* emergence, but its moderate use is acceptable for this parasitoid. Diflubenzuron had severe transgenerational side-effects. Tebuconazole fungicide and triflumuron insecticide are compatible with *P. elaeisis* in sustainable integrated pest management (IPM) programs, while malathion can not be included in them.

## Introduction

Beneficial arthropods provide ecological services such as dung burial, pest control and pollination^1^. However, pesticide applications may have direct or indirect negative effects on these non-target organisms^2, 3^, reducing the strength of their ecological services^4^.

The significant contribution of parasitoids to pest control is due to their high host specificity^5^, but generalist ones can contribute to the control of arthropod pests. In Brazil, management of soybean (*Glycine max* (L.) Merrill) pests includes biological control^6^. *Palmistichus elaeisis* Delvare and LaSalle, 1993 (Hymenoptera: Eulophidae) is a generalist and gregarious endoparasitoid of Coleoptera and Lepidoptera pupae^7^. This parasitoid is released to control *Anticarsia gemmatalis* Hubner (Lepidoptera: Noctuidae) in soybean crops^8^, however, pesticides are still widely used in this crop^6^.

Natural enemies of insects are susceptible to pesticides^9^, which may disrupt their efficiency for biological control^10, 11^. Organophosphates are broad-spectrum insecticides with high toxicity for non-target arthropods^12^; some herbicides have shown side-effects on different non-target organisms^13–15^. Fungicides are generally non-toxic for insects, but may have synergistic effects with other pesticides^16^; while growth regulator insecticides (GRI) are safer for biological control agents^5^, but can have lethal and sublethal effects on immature stages or on reproduction^17^.

New plant protection strategies focus on minimizing chemical pesticide use, and seek its compatibility with biological control agents^18^. The low toxicity of pesticides for non-target arthropods is an important component for sustainable integrated pest management (IPM)^19^. Parasitoids and predators can be exposed directly to pesticides during pesticide applications or, more commonly, indirectly by host hemolymph and contaminated prey tissues^11^. In this way, these insects remain exposed to sublethal doses for longer time periods than lethal doses^19^. The exposure to pesticides does not necessarily result in the death of natural enemies^20^, but their effects may comprise the development, longevity and fecundity of these insects, as well as behaviors involved in mobility, foraging for hosts (or prey) and mates^17^.

In Brazil, glyphosate-resistant (GR) soybean varieties represent 86% of the cultivated area, making the use of this herbicide for weed management inevitable^21^. Soybean rust (*Phakopsora pachyrhizi* Sid.) is a devastating foliar disease of this crop^22^, and the fungicide tebuconazole is widely used to control this disease. IPM programs established in soybean crops in Brazil, have allowed an over 50% reduction in insecticide use^23^. However, *A. gemmatalis* infestations sometimes require the application of insecticides. Among the most used are the GRI’s diflubenzuron and triflumuron, but when infestations are high density, contact insecticides such as malathion are applied^23^. Knowledge of the side-effects of these pesticides on *P. elaeisis* is scarce, and requires further research.

The objective of this study was to evaluate the side-effects of diflubenzuron, glyphosate, malathion, tebuconazole and triflumuron, pesticides used for soybean crops, on the performance of *P. elaeisis* fed with *A. gemmatalis* pupae from caterpillars exposed to these pesticides.

## Results

*Palmistichus elaeisis* was exposed indirectly to pesticides, authorized for soybean cultivation in Brazil, through treated hosts. *Anticarsia gemmatalis* caterpillars were fed an artificial diet or soybean leaves treated with glyphosate and/or tebuconazole to obtain the pupae. Additionally, *A. gemmatalis* pupae from caterpillars fed only an artificial diet (non-treated) were immersed in different pesticide solutions (Table 1).

**Table 1:** Pesticides evaluated on the parasitoid *Palmistichus elaeisis* (Hymenoptera: Eulophidae) exposed to *Anticarsia gemmatalis* (Lepidoptera: Noctuidae) pupae treated with them. These pesticides are authorized for use in soybean cultivation in Brazil.

Trade name^a^	Pesticide type	Active ingredient (g ai L^−1^)	Field dose (g ai ha^−1^)	TC-EC^b^
Control (water)	—	—	—	—
Roundup Ready^®^^c^	Herbicide	Glyphosate (480)	720	II-III
Folicur 200 CE^®^	Fungicide	Tebuconazole (200)	150	III-II
Dimilin 80 WG^®^	Insecticide	Diflubenzuron (800)	30–60 (45)^d^	III-III
Certero^®^	Insecticide	Triflumuron (480)	14–24 (20)^d^	IV-III
Malathion Chab^®^	Insecticide	Malathion (500)	400	III-III

^a^Pesticide manufacturers: Roundup Ready^®^, Monsanto do Brasil Ltda, São José dos Campos, São Paulo, Brazil; Folicur 200 CE^®^ and Certero^®^, Bayer SA, Bayer CropScience Brasil, São Paulo, Brazil; Dimilin 80 WG^®^, Arysta Lifescience do Brasil Ind. Quím. e Agropec. Ltda, Salto de Pirapora, São Paulo, Brazil; Malathion Chab^®^, Cheminova Brasil Ltda, Goiânia, Goiás, Brazil. Mention of trade names or commercial products in this publication is solely for the purpose of providing specific information and does not imply their recommendation. ^b^TC = Toxicological Class: I, extremely toxic; II, highly toxic; III, moderately toxic; IV, slightly toxic, and EC = Environmental Classification: I, highly dangerous; II, very dangerous; III, dangerous; IV, low dangerous. ^c^Glyphosate concentrations given as g acid equivalent of [N- (phosphonomethyl) glycine]. ^d^Dose used in this work.

### Caterpillars feed a treated artificial diet

*Palmistichus elaeisis* presented high parasitism and emergence rates from *A. gemmatalis* pupae (>85%). Glyphosate increased the number of parasitoids emerged and females produced per female with 164 and 25 individuals, respectively. This herbicide and tebuconazole did not affect the other *P. elaeisis* reproductive parameters (Table 2).

**Table 2:** *Palmistichus elaeisis* (Hymenoptera: Eulophidae) reproductive parameters reared on *Anticarsia gemmatalis* (Lepidoptera: Noctuidae) pupae. Caterpillars of *A. gemmatalis* were fed with artificial diet treated with glyphosate and tebuconazole.

Reproductive parameters	Water	Glyphosate	Tebuconazole
Life cycle duration (days)^ns^	23.5 ± 0.3	23.6 ± 0.5	22.8 ± 0.4
Parasitism (%)^ns^	100.0	100.0	93.8
Emergence (%)^ns^	87.5	93.8	87.5
Total progeny	131 ± 10^b^	164 ± 16^a^	119 ± 13^b^
Females produced per female	19.9 ± 1.5^b^	24.8 ± 2.4^a^	18.3 ± 1.7^b^
Female cephalic capsule width (mm)^ns^	0.60 ± 0.01	0.59 ± 0.01	0.58 ± 0.01
Male cephalic capsule width (mm)^ns^	0.47 ± 0.01	0.48 ± 0.01	0.48 ± 0.01
Females longevity (days)^ns^	33.1 ± 3.2	30.6 ± 3.0	31.6 ± 2.9
Male longevity (days)^ns^	31.9 ± 3.2	29.9 ± 2.9	30.7 ± 3.4
Sex ratio^ns^	0.91 ± 0.01	0.91 ± 0.01	0.89 ± 0.01
Host pupa weight (mg)	223 ± 7	220 ± 9	214 ± 9

Means with the same letter per line do not differ at 5% probability by Tukey test. ^ns^non significant at 5% probability. ± Standard error (n = 16).

### Caterpillars feed with treated soybean leaves

The parasitism rate of *P. elaeisis* (>80%) on *A. gemmatalis* pupae, from caterpillars fed with soybean leaves treated with glyphosate and tebuconazole, was similar to that of the control. However, the emergence of this parasitoid was lower than 50% from pupae in the treatment with soybean leaves treated with glyphosate. *Palmistichus elaeisis* emergence was lower from *A. gemmatalis* pupae whose caterpillars were fed with leaves from the GR soybean cultivar CD214-RR with or without glyphosate treatment. *Anticarsia gemmatalis* pupae fed with this cultivar also had the lowest weight, and the cephalic capsule width and longevity of *P. elaeisis* females was smaller than other treatments, possibly due to poor nutrition of this parasitoid with these smaller size pupae (Table 3).

**Table 3:** *Palmistichus elaeisis* (Hymenoptera: Eulophidae) reproductive parameters reared on *Anticarsia gemmatalis* (Lepidoptera: Noctuidae) pupae. Caterpillars of *A. gemmatalis* were fed with leaves of different soybean cultivars immersed in glyphosate and tebuconazole. Leaves of the controls were immersed in water.

Reproductive parameters	Treatment^a^/Soybean cultivar							
	Control		Glyphosate		Control		Tebuconazole	
	CD212-RR	CD214-RR	CD212-RR	CD214-RR	OC14	CD201	OC14	CD201
Life cycle duration (days)^ns^	19.8 ± 1.4	19.8 ± 0.8	20.3 ± 1.5	19.4 ± 0.7	20.9 ± 0.4	21.8 ± 1.4	20.3 ± 0.4	20.6 ± 0.4
Parasitism (%)^ns^	93.8	81.3	81.3	93.8	100.0	93.8	81.3	93.8
Emergency (%)^b^	75^a^	68.8^c^	37.5^d^	31.3^e^	68.8^b^	75.0^a^	62.5^c^	68.8^b^
Total progeny^ns^	119 ± 26	99 ± 22	163 ± 78	101 ± 28	126 ± 19	120 ± 16	165 ± 14	133 ± 16
Females produced per female^ns^	12.7 ± 2.8	11.8 ± 3.3	20.7 ± 10.9	8.0 ± 2.0	11.7 ± 2.4	13.1 ± 3.1	18.4 ± 3.3	13.5 ± 2.4
Female cephalic capsule width (mm)^ns^	0.56 ± 0.01	0.53 ± 0.02	0.56 ± 0.02	0.53 ± 0.01	0.56 ± 0.01	0.55 ± 0.01	0.53 ± 0.01	0.54 ± 0.02
Male cephalic capsule width (mm)^c^	0.46 ± 0.01^a^	0.42 ± 0.02^b^	0.43 ± 0.01^a,b^	0.42 ± 0.01^b^	0.44 ± 0.01^a,b^	0.45 ± 0.01^a,b^	0.44 ± 0.01^a,b^	0.44 ± 0.01^a,b^
Female longevity (days)^c^	37.2 ± 2.9^a,b^	30.8 ± 2.5^b^	34.6 ± 3.6^a,b^	28.8 ± 2.9^b^	40.8 ± 2.3^a^	36.3 ± 2.9^a,b^	35.9 ± 3.2^a,b^	34.3 ± 3.5^a,b^
Male longevity (days)^ns^	38.0 ± 3.2	37.1 ± 3.7	30.8 ± 5.6	34.4 ± 5.7	39.4 ± 3.8	38.6 ± 3.2	33.4 ± 3.9	36.6 ± 4.0
Sex ratio^ns^	0.72 ± 0.04	0.67 ± 0.05	0.70 ± 0.07	0.64 ± 0.07	0.72 ± 0.06	0.72 ± 0.04	0.63 ± 0.08	0.65 ± 0.1
Host pupa weight (mg)^c^	208 ± 9^a,b^	169 ± 8^c^	219 ± 10^a^	170 ± 5^c^	213 ± 9^a,b^	195 ± 9^b^	226 ± 7^a^	215 ± 9^a,b^

^a^The pesticide solutions of each treatment were prepared according to the manufacturer instructions at the recommended field doses. ^b^Means with the same letter per line do not differ at 5% probability by Kruskal-Wallis test. ^c^Means with the same letter per line do not differ at 5% probability by Tukey test. ^ns^non-significant at 5% probability. ± Standard error (n = 16).

### Pupae immersed in pesticide solutions

*Palmistichus elaeisis* died before parasiting *A*. *gemmatalis* pupae treated with malathion. Parasitism rates of *P*. *elaeisis* were 58.3, 83.3, 100, 100 and 100% with triflumuron, diflubenzuron, tebuconazole, glyphosate and the control, respectively, with an emergence rate of 50% or higher in all treatments without differences between them. Total number of parasitoids emerged per host ranged from 112 to 195. Glyphosate and triflumuron presented the highest progeny rates with 194 and 195 individuals, respectively. Longevity ranged from 30.0 to 40.6 days. The longevity of *P. elaeisis* females from *A. gemmatalis* pupae immersed in triflumuron solution was longer than that of the control. The other *P. elaeisis* reproductive parameters were similar between treatments, except for the malathion (Table 4).

**Table 4:** *Palmistichus elaeisis* (Hymenoptera: Eulophidae) reproductive parameters reared on *Anticarsia gemmatalis* (Lepidoptera: Noctuidae) pupae immersed in different pesticide solutions. Pupae from the controls were immersed in water.

Reproductive parameters	Treatments^a^					
	Control	Glyphosate	Tebuconazole	Diflubenzuron	Triflumuron	Malathion
Life cycle duration (days)^ns^	20.3 ± 0.5	19.1 ± 0.3	20.3 ± 0.6	20.2 ± 0.6	18.5 ± 0.3	ND
Parasitism (%)^b^	100.0^a^	100.0^a^	100.0^a^	83.3^b^	58.3^c^	0.0^d^
Emergence (%)^ns^	66.7	91.7	66.7	50.0	50.0	ND
Total progeny^c^	141 ± 22^b^	194 ± 16^a^	124 ± 19^b^	112 ± 34^b^	195 ± 32^a^	ND
Females produced per female^ns^	18.3 ± 3.2	23.1 ± 2.7	17.3 ± 2.4	20.2 ± 4.3	26.4 ± 5.7	ND
Female cephalic capsule width (mm)^ns^	0.57 ± 0.01	0.56 ± 0.01	0.56 ± 0.02	0.59 ± 0.01	0.56 ± 0.01	ND
Male cephalic capsule width (mm)^ns^	0.46 ± 0.01	0.44 ± 0.01	0.44 ± 0.01	0.47 ± 0.03	0.44 ± 0.01	ND
Females longevity (days)^c^	30.5 ± 3.2^b^	36.9 ± 4.3^a,b^	30.0 ± 3.8^b^	35.9 ± 2.4^a,b^	40.6 ± 3.8^a^	ND
Male longevity (days)^ns^	40.1 ± 7.4	39.2 ± 6.9	35.3 ± 6.0	36.9 ± 5.3	32.8 ± 6.2	ND
Sex ratio^ns^	0.82 ± 0.06	0.82 ± 0.04	0.81 ± 0.07	0.87 ± 0.08	0.79 ± 0.05	ND

^a^The pesticide solutions of each treatment were prepared according to the manufacturer instructions at the recommended field doses. ^b^Means with the same letter per line do not differ at 5% probability by Kruskal-Wallis test. ^c^Means with the same letter per line do not differ at 5% probability by Tukey test. ^ns^not significant at 5% probability. ND = non-determinated. ± Standard error (n = 16).

Parasitism of *P*. *elaeisis* descendants, from individuals reared on *A. gemmatalis* pupae immersed in diflubenzuron and triflumuron solutions, was higher than 90% with a rate of emergence of 58%. Progeny of this parasitoid was higher with diflubenzuron and triflumuron, however, most individuals with the first insecticide were males (135) with only six females per female being produced. This treatment presented the lowest sex ratio in the second *P. eleasis* generation (Table 5).

**Table 5:** Reproductive parameters of the second generation *Palmistichus elaeisis* (Hymenoptera: Eulophidae) from individuals, reared on *Anticarsia gemmatalis* (Lepidoptera: Noctuidae) pupae immersed in diflubenzuron and triflumuron solutions. *Anticarsia gemmatalis* pupae use to rear the second *P. elaeisis* generation were obtained from caterpillars fed only with artificial diet.

Reproductive parameters	Control	Diflubenzuron	Triflumuron
Life cycle duration (days)^ns^	21.6 ± 0.5	20.4 ± 0.4	20.6 ± 0.3
Parasitism (%)^ns^	100	100	91.0
Emergence (%)^ns^	58.3	58.3	58.3
Total progeny^ns^	111 ± 18^b^	171 ± 26^a^	163 ± 9^a^
Females produced per female	12.0 ± 2.0^a^	6.0 ± 2.0^b^	14.3 ± 2.3^a^
Total females	72 ± 11^a^	36 ± 12^b^	94 ± 14^a^
Total males	39 ± 12^b^	135 ± 18^a^	69 ± 13^b^
Sex. ratio	0.64 ± 0.07^a^	0.21 ± 0.04^b^	0.57 ± 0.08^c^

Means with the same letter per line do not differ at 5% probability by Tukey test. ^ns^not significant at 5% probability. ± Standard error (n = 16).

## Discussion

Exposure to extreme environmental conditions^24^, toxic compounds^25^, and host type^26^ may affect insect reproductive traits and its longevity. *Palmistichus elaeisis* performance in the controls, artificial diet and soybean leaves used to feed *A. gemmatalis* caterpillars to obtain the pupae, indicated their compatibility for mass rearing of this parasitoid as found in others studies^27^, except for the GR soybean cultivar CD214-RR that resulted in a poor food source. *Anticarsia gemmatalis* caterpillars of this lepidopteran fed with this cultivar produced smaller pupae, which reduced the *P. elaeisis* female performance. The smaller cephalic capsule width and the lower longevity and number of females produced per female with this soybean cultivar, can be explained by the lack of space inside the host leading to intraspecific competition^7, 28^. Plants can affect host development and parasitoid population ecology^29^. Low quality of the GR soybean cultivar CD214-RR as a food source can not be attributed to the gene endowing glyphosate resistance, because transgenic crops do not have side-effects on non-target organisms^30^. In addition, the other GR cultivar (CD212-RR), was a good food source for *A. gemmatalis*.

High parasitism and emergence rates of *P. elaeisis* from *A. gemmatalis* pupae, whose caterpillars were fed an artificial diet immersed in glyphosate and tebuconazole solutions, shows the safety of these pesticides for this parasitoid. The greater number of progeny of this parasitoid with glyphosate may be due to this herbicide stimulating oviposition. This side-effect of glyphosate was observed in *Polyphagotarsonemus latus* Banks (Acari: Tarsonemidae), and *Tetranychus bastosi* (Tuttle) Baker and Sales (Acari: Tetranychidae) when exposed at 360 g ae/ha^14^. Effects of this herbicide vary between species^31^, and had contrasting effects on foraging behavior of the predator spiders *Tigrosa helluo* Walckenaer and *Pardosa milvina* Hentz (Araneae: Lycosidae)^13^; caused early activation of anantioxidant defense in *Drosophila melanogaster* Meigen (Diptera: Drosophilidae)^31^; and severely reduced the reproduction and fecundity of *Chrysoperla externa* Hagen (Neuroptera: Chysopidae)^15^, but improved this parameter for *P. latus* and *T. bastosi* ^14^. Effects of glyphosate-based herbicides could be mainly associated with chemicals (surfactants, adjuvants and others) not specified on the label. Different glyphosate formulations decreased the parasitism and egg viability rates of *Telenomus remus* Nixon (Hymenoptera: Platygastridae) between 20 to 75%^32^. In addition, detrimental effects of glyphosate are more likely to occur over the long term with continuous use of this herbicide^15^. This explains the low *elaeisis* emergence from *A. gemmatalis* pupae, whose caterpillars were exposed to glyphosate each time that soybean leaves were offered *ad libitum* (3–5 times). Glyphosate use in the field is acceptable for the performance of this parasitoid, because applications of this herbicide for weed control are not frequent over short time periods.

The lack of tebuconazole side-effects on the development, reproduction and longevity of *P. eleais* agrees with findings from the “International Organization for Biological Control–West Palaearctic Regional Section (IOBC/WPRS)”-Working Group “Pesticides and Beneficial Organisms”, indicating that this fungicide is not harmful to parasitoids and predators^33^. In addition, this compound is an antifungal agent for rearing lepidopteran larvae on an artificial diet^34^. However, a synergistic effect of this fungicide with other pesticides^16^, can not be discounted.

Total *P. elaeisis* mortality by malathion contact or inhalation before parasitism showed the lethal impact of this broad-spectrum insecticide. Mortality by malathion is attributed to its rapid transformation to oxygenases enzymes, to malaoxon and isomalathion inhibiting the acetylcholinesterase and other enzymatic systems functioning in its detoxification^12^. *Palmistichus elaeisis* can not detoxify the malathion but this insecticide presents low specificity and may attract natural enemies^35^. Malathion did not cause mortality of *A. gemmatalis* pupae, possibly due to its rapid degradation^36^, and to the cuticular protection reducing insecticide uptake.

The GRI’s, diflubenzuron and triflumuron, did not affect the population dynamics of the first *P. elaeisis* generation, and the parasitism and emergence of the second. However, diflubenzuron reduced the sex ratio of individuals from the second generation. This could be due to reduced sperm production or male sterility, since some hymenopterans have arrenotocal parthenogenesis where males develop from unfertilized eggs and females from fertilized ones^37^. In addition, this GRI can have ovicidal activity leading to a reduction in the reproduction rates of this parasitoid^38^. The possible sterility of *P. elaeisis* males from diflubenzuron exposure from the first generation onwards requires further investigation, because IGR side-effects are different depending on species^39^. The sex ratio and longevity of the parasitoid *Tamarixia radiata* Waterston (Hymenoptera: Eulophidae) was not affected with diflubenzuron^40^, but this insecticide had an indirect impact on the reproduction and population dynamics of the predator *Podisus nigripinus* Dallas (Hemiptera: Pentatomidae)^38^. Diflubenzuron and pyriproxyfen delayed the development from egg to pupa, emergence of adults and reduced the lifespan of *Hyposoter didymator* Thunberg (Hymenoptera: Ichneumonidae) females of the F_2_ generations, demonstrating the transgenerational effects of these GRI’s^41^. Chlorfluazuron inhibited testicular development and spermatogenesis of *Spodoptera litura* F. (Lepidoptera: Noctuidae) males^42^. *Anagrus nilaparvatae* Pang et Wang (Hymenoptera: Mymaridae) adults exposed to chlorfluazuron had no mortality, but its fertility and the longevity of females was reduced^43^. Evaluating the pesticide effects in more than one generation, provides an understanding of the unidentified effects in a first analysis^4^. Transgenerational effects caused by diflubenzuron on reproductive *P. elaeisis* parameters, even without exposing the second generation to the insecticide, strongly suggests that direct contact in the field could have lethal effects on this parasitoid, since first generation individuals were exposed only through indirect contact from a treated host.

The low toxicity of triflumuron for *P. elaeisis* development may be related to its action mechanism as a chitin synthesis inhibitor, but with little impact on beneficial insects, with low absorption through ingestion by these insects^44^. Triflumuron did not affect the sex ratio of the second generation of *Trichogramma galloi* Zucchi (Hymenoptera: Trichogrammatidae)^5^, and others GRI’s are also harmless for non-target arthropods such as spirotetramat for *Chrysoperla carnea* Stephens (Neuroptera: Chrysopidae) and *Adalia bipunctata* L. (Coleoptera: Coccinellidae) larvae and adults^45^; methoxyfenozide for several non-target arthropods^2^; and tebufenozide, hexaflumuron, and tebufenozide for *Trichogramma* species adults^5, 46, 47^.

## Conclusions

The herbicide glyphosate and the insecticide diflubenzuron reduced the performance of *P. elaeisis*. Continuous exposure of the host to glyphosate may represent long-term risks, but a responsible use of this herbicide is acceptable. Diflubenzuron had transgenerational side-effects in the reproductive performance of the second *P. elaeisis* generation, even without exposing this parasitoid to the insecticide. Direct contact of diflubenzuron could have lethal effects on *P. elaeisis*. The fungicide tebuconazole and the insecticide triflumuron are compatible with *P. elaeisis* in IPM programs, and malathion can not be included in these.

## Material and Methods

### Biological Material

#### Anticarsia gemmatalis

Caterpillars of the pest were obtained from the Laboratory of Biological Control of Insects (LCBI-BIOAGRO) at the Universidade Federal de Viçosa (UFV), where this insect is reared on an artificial diet^48^, with 125 g bean, 62.4 g beer yeast, 100 g wheat germ, 100 g soy protein, 50 g casein, 35 g agar, 5 g nipagin, 6 g ascorbic acid, 3 g sorbic acid, 6 mL formol at 40% in water, and 10 mL vitaminic solution. Second instar caterpillars were reared in 500 mL plastic containers, receiving soybean leaves *ad libitum* (3–5 times) or an artificial diet according to the treatments until pupae stage. Plastic pots were kept in an acclimatized chamber at 25 ± 2 °C, 70 ± 10% relative humidity and 14:10 h (light:dark) photoperiod.

#### Palmistichus elaeisis

Adults were kept in glass tubes (14.0 × 2.2 cm) with honey droplets as food source. The tubes were closed with a cotton plug. *Anticarsia gemmatalis* pupae with 48–72 h-old  were exposed to *P. elaeisis* females for 24 h at a density of 6:1 females:pupae^48^, in the same rearing conditions as for *A. gemmatalis*. These new offspring were used in the experiments.

#### Soybean

Two GR cultivars (CD212-RR and CD214-RR) and two conventional ones (CD201 and OC14) were used. The four cultivars were obtained from Coodetec (Cooperativa Central de Pesquisa Agrícola Ltda, Rio Verde, Goiás, Brazil). Five soybean seeds were planted per pot (3 L) with substrate (soil: organic matter) fertilized with ammonium sulphate (equivalent to 50 kg N ha^−1^). These pots were kept in the greenhouse of the Soybean Breeding Program at the UFV in Viçosa, Minas Gerais, Brazil, and the plants were watered daily. Three plants with 3–4 true leaves were kept after germination and used from V6 to R6 phenological stages^49^.

### *Palmistichus elaeisis* performance when treating the host with pesticides

The pesticides tested are authorized for use in soybean cultivation in Brazil, and they were prepared at the recommended field doses (Table 1).

#### Caterpillars fed an artificial diet treated with glyphosate

The artificial diet was immersed in glyphosate solution or water (control) for 5 s, and offered to *A. gemmatalis* carterpillars from the second to the fifth instar.

#### Caterpillars fed with treated soybean leaves

Soybean leaves were immersed in the corresponding pesticide solution for 5 s, and offered *ad libitum* (3–5 times) to *A. gemmatalis* caterpillars from the second to the fifth instar. Control leaves were immersed in water. Because this lepidopteran can have different preference levels for soybean cultivars as a source of food^50^, two GR **c**ultivars (CD212-RR and CD214-RR) were used for glyphosate, and two conventional ones (CD201 and OC14) for tebuconazole, so that this factor does not alter the interpretation of our results.

#### Pupae immersed in pesticide solutions

*Anticarsia gemmatalis* pupae from caterpillars fed an artificial diet were immersed quickly (5 s) in the different pesticide (glyphosate, tebuconazole, diflubenzuron, triflumuron, malathion) solutions or water (control).

Growth regulator insecticides are related to transgenerational side-effects on reproduction of non-target arthropods^17^, therefore, performance of second *P. elaeisis* generation with GRI’s, diflubezuron and triflumuron, was studied. *Anticarsia gemmatalis* pupae from caterpillars fed only on an artificial diet were used, and were not submerged in insecticide solutions.

In all experiments, 48–72 h-old *A. gemmatalis* pupae, obtained from caterpillars fed an artificial diet or soybean leaves treated with pesticides according to the respective treatments described previously, were exposed to 48–72 h-old *P. elaeisis* females for 48 h. Each experimental unit consisted of one pupa for every six *P. elaeisis* females (1:6 pupae:females) placed into glass tubes (14.0 × 2.2 cm). The experiments were conducted in a completely random design with 16 replications, and glass tubes were kept in an acclimatized chamber at 25 ± 2 °C, 70 ± 10% relative humidity with a 14:10 h (light:dark) photoperiod.

Life cycle duration of *P. elaeisis* (egg to adult), parasitism percentage not considering natural host mortality^51^, emergence percentage of progeny, and the cephalic capsule width (mm) of the parasitoids emerged from each *A. gemmatalis* pupae were evaluated. The cephalic capsule was measured with a micrometric ocular in a stereoscopic microcopy. Sex ratio was calculated as *Rs = female number/total parasitoid number*. Individuals were sexed according to the antenna and abdomen morphological characteristics of this parasitoid^52^.

### Statistics analysis

*Anticarsia gemmatalis* pupae weight and *P. elaeisis* reproductive parameters were submitted to ANOVA. Statistical analysis was performed with Statistix software version 9.0 (Analytical Software, USA). The means were compared using Tukey’s test at 95% probability level when necessary. *Palmistichus elaeisis* parasitism and emergence (%) were submitted to non-parametric analysis at 95% probability.

## References

[1] Kaur-Gill, H. & Garg, H. Pesticides: Environmental impacts and management strategies in *Pesticides - Toxic Aspects* (eds Larramendy, M. L. & Soloneski, S.) 187–230 (Intech, 2014).

[2] Loetti, V. & Bellocq, I. Effects of the insecticides methoxyfenozide and cypermethrin on non-target arthropods: A field experiment. *Aust. Entomol*. **56**, 255–260 (2017).

[3] Tavares WS (2010). Selective effects of natural and synthetic insecticides on mortality of *Spodoptera frugiperda* (Lepidoptera: Noctuidae) and its predator *Eriopis connexa* (Coleoptera: Coccinellidae). J. Environ. Sci. Health B.

[4] Biondi A, Zappalà L, Stark JD, Desneux N (2013). Do biopesticides affect the demographic traits of a parasitoid wasp and its biocontrol services through sublethal effects?. PLoS One.

[5] Costa MA (2014). Sublethal and transgenerational effects of insecticides in developing *Trichogramma galloi* (Hymenoptera: Trichogrammatidae). Ecotoxicology.

[6] Castro AA (2015). Demographic parameters of the insecticide-exposed predator *Podisus nigrispinus*: implications for IPM. BioControl.

[7] Pereira, K.S., Guedes, N. M. P., Serrão, J. E., Zanuncio, J. C. & Guedes, R. N. C. Superparasitism, immune response and optimum progeny yield in the gregarious parasitoid *Palmistichus elaeisis*. *Pest Manag. Sci*. **73**, 1101–1109 (2017). 10.1002/ps.4534 28127857

[8] Pereira FF (2013). Biological characteristics of *Palmistichus elaeisis* Delvare & LaSalle (Hymenoptera: Eulophidae) on refrigerated pupae of *Anticarsia gemmatalis* Hubner (Lepidoptera: Noctuidae). Chil. J. Agric. Res..

[9] Zanuncio JC, Batalha VC, Guedes RNC, Picanço MC (1998). Insecticide selectivity to *Supputius cincticeps* (Stal) (Het., Pentatomidae) and its prey *Spodoptera frugiperda* (J. E. Smith) (Lep., Noctuidae). J. Appl. Entocol..

[10] Biondi A (2015). Life stage-dependent susceptibility of *Aphytis melinus* DeBach (Hymenoptera: Aphelinidae) to two pesticides commonly used in citrus orchards. Chemosphere.

[11] Addison PJ, Barker GM (2006). Effect of various pesticides on the non-target species *Microctonus hyperodae*, a biological control agent of *Listronotus bonariensis*. Entomol. Exp. Appl..

[12] Büyükgüzel K (2006). Malathion-induced oxidative stress in a parasitoid wasp: effect on adult emergence, longevity, fecundity, and oxidative and antioxidative response of *Pimpla turionellae* (Hymenoptera: Ichneumonidae). J. Econ. Entomol..

[13] Rittman S, Wrinn KM, Evans SC, Webb AW, Rypstra AL (2013). Glyphosate-based herbicide has contrasting effects on prey capture by two co-occurring wolf spider species. J. Chem. Ecol..

[14] Saraiva AS (2016). Glyphosate sub-lethal toxicity to non-target organisms occurring in *Jatropha curcas* plantations in Brazil. Exp. Appl. Acarol..

[15] Schneider MI, Sanchez N, Pineda S, Chi H, Ronco A (2009). Impact of glyphosate on the development, fertility and demography of *Chrysoperla externa* (Neuroptera: Chrysopidae). Ecological approach. Chemosphere.

[16] Papaefthimiou C, Theophilidis G (2001). The cardiotoxic action of the pyrethroid insecticide deltamethrin, the azole fungicide prochloraz, and their synergy on the semi-isolated heart of the bee *Apis mellifera macedonica*. Pest. Biochem. Physiol..

[17] Desneux N, Decourtye A, Delpuech JM (2007). The sublethal effects of pesticides on beneficial arthropods. Annu. Rev. Entomol..

[18] Gradish AE, Scott-Dupree CD, Shipp L, Harris CR, Ferguson G (2011). Effect of reduced risk pesticides on greenhouse vegetable arthropod biological control agents. Pest Manag. Sci..

[19] Stanley, J. & Preetha, G. Pesticide toxicity to arthropod predators: Exposure, toxicity and risk assessment methodologies in *Pesticide toxicity to non-target organisms* (eds. Stanley, J. & Preetha, G.) 1–98 (Springer Netherlands, 2016).

[20] Castro AA (2013). Survival and behavior of the insecticide-exposed predators *Podisus nigrispinus* and *Supputius cincticeps* (Heteroptera: Pentatomidae). Chemosphere.

[21] Hungria M (2014). Effects of the glyphosate-resistance gene and herbicides on soybean: Field trials monitoring biological nitrogen fixation and yield. Field Crops Res..

[22] Twizeyimana M, Hartman GL (2017). Sensitivity of *Phakopsora pachyrhizi* isolates to fungicides and reduction of fungal infection based on fungicide and timing of application. Plant Dis..

[23] Panizzi AR (2013). History and contemporary perspectives of the integrated pest management of soybean in Brazil. Neotrop. Entomol..

[24] Abbes K (2015). Combined non-target effects of insecticide and high temperature on the parasitoid *Bracon nigricans*. PLoS One.

[25] Zanuncio JC, Saavedra JLD, Oliveira HN, De Ghelle D, De Clercq O (1996). Development of the predatory stinkbug *Brontocoris tabidus* Signoret Heteroptera: Pentatomidae on different proportions of an artificial diet and pupae of *Tenebrio molitor* L. Coleoptera: Tenebrionidae. Biocontrol Sci. Technol..

[26] Benelli, G. *et al*. The impact of adult diet on parasitoid reproductive performance. *J. Pest Sci*. **90**; doi:10.1007/s10340-017-0835-2 (2017).

[27] Zanuncio JC, Pereira FF, Jacques GC, Tavares MT, Serrão JE (2008). *Tenebrio molitor* Linnaeus (Coleoptera: Tenebrionidae), a new alternative host to rear the pupae parasitoid *Palmistichus elaeisis* Delvare & LaSalle (Hymenoptera: Eulophidae). Coleopt. Bull..

[28] Jervis MA, Ellers J, Harvey JA (2008). Resource acquisition, allocation, and utilization in parasitoid reproductive strategies. Annu. Rev. Entomol..

[29] Bukovinszky T, F van Veen FJ, Jongema Y, Dicke M (2008). Direct and indirect effects of resource quality on food web structure. Science.

[30] Klümper W, Qaim M (2014). A meta-analysis of the impacts of genetically modified crops. PLoS One.

[31] Aguiar LM, Figueira FF, Gottschalk MS, Rosa CE (2016). Glyphosate-based herbicide exposure causes antioxidant defence responses in the fruit fly *Drosophila melanogaster*. Comp. Biochem. Physiol. C Toxicol. Pharmacol..

[32] Stecca CS (2016). Side-effects of glyphosate to the parasitoid *Telenomus remus* Nixon (Hymenoptera: Platygastridae). Neotrop. Entomol..

[33] Sterk G (1999). Results of the seventh joint pesticide testing programme carried out by the IOBC/WPRS-Working Group ‘Pesticides and Beneficial Organisms’. BioControl.

[34] Su J, Wang YC, Zhang SK, Ren XB (2014). Antifungal agents against *Aspergillus niger* for rearing rice leaffolder larvae (Lepidoptera: Pyralidae) on artificial diet. J. Econ. Entomol..

[35] Rao A, Vinson SB, Gilstrap FE, Michels GL (1999). Response of an aphid parasitoid, *Aphelinus asychis* to its host, plant, host-plant complex, and to malathion. Entomol. Exp. Appl..

[36] Childers CC, Villanueva R, Aguilar H, Chewning R, Michaud JP (2001). Comparative residual toxicities of pesticides to the predator Agistemus industani (Acari: Stigmaeidae) on citrus in Florida. Exp. Appl. Acarol..

[37] Rabeling C, Kronauer DJC (2013). Thelytokous parthenogenesis in eusocial Hymenoptera. Annu. Rev. Entomol..

[38] Castro AA, Lacerda MC, Zanuncio TV (2012). Effect of the insect growth regulator diflubenzuron on the predator *Podisus nigrispinus* (Heteroptera: Pentatomidae). Ecotoxicology.

[39] Campiche S, Slooten KB, Ridreau C, Tarradellas J (2006). Effects of insect growth regulators on the non-target soil arthropod *Folsomia candida* (Collembola). Ecotoxicol. Environ. Saf..

[40] Lira ACS (2015). Lethal and sublethal impacts of acaricides on *Tamarixia radiata* (Hemiptera: Eulophidae), an important ectoparasitoid of *Diaphorina citri* (Hemiptera: Liviidae). J. Econ. Entomol..

[41] Schneider M, Smagghe G, Pineda S, Viñuela E (2008). The ecological impact of four IGR insecticides in adults of *Hyposoter didymator* (Hym, Ichneumonidae): pharmacokinetics approach. Ecotoxicology.

[42] Perveen F (2008). Effects of sublethal doses of chlorfluazuron on insemination and number of inseminated sperm in the common cutworm, *Spodoptera litura* (F.) (Lepidoptera: Noctuidae). Entomol. Sci..

[43] Wang HY (2008). Assessment of the impact of insecticides on *Anagrus nilaparvatae* (Pang et Wang) (Hymenoptera: Mymaridae), an egg parasitoid of the rice planthopper, *Nilaparvata lugens* (Hemiptera: Delphacidae). Crop. Prot..

[44] Merzendorfer H (2013). Chitin synthesis inhibitors: old molecules and new developments. Insect Sci..

[45] Garzón A, Medina P, Amor F, Viñuela E, Budia F (2015). Toxicity and sublethal effects of six insecticides to last instar larvae and adults of the biocontrol agents *Chrysoperla carnea* (Stephens) (Neuroptera: Chrysopidae) and *Adalia bipunctata* (L.) (Coleoptera: Coccinellidae). Chemosphere.

[46] Wang Y (2012). Insecticide toxic effects on *Trichogramma ostriniae* (Hymenoptera: Trichogrammatidae). Pest Manag. Sci..

[47] Wang Y (2014). Toxicity risk of insecticides to the insect egg parasitoid *Trichogramma evanescens* Westwood (Hymenoptera: Trichogrammatidae). Pest Manag. Sci..

[48] Pastori PL (2012). Density of females of *Palmistichus elaeisis* Delvare & LaSalle, 1993 (Hymenoptera: Eulophidae) for reproduction in *Anticarsia gemmatalis* Hübner, 1818 (lepidoptera: noctuidae) pupae. Arq. Inst. Biol..

[49] Casteel, S. N. Soybean physiology: How well do you know soybeans? *Soybean Station* https://www.agry.purdue.edu/ext/soybean/Arrivals/10SoyDevt.pdf (2010).

[50] Fugi CGQ, Lourenção AL, Parra JRP (2005). Biology of *Anticarsia gemmatalis* on soybean genotypes with different degrees of resistance to insects. Sci. Agric..

[51] Abbot WS (1925). A method of computing the effectiveness of an insecticide. J. Econ. Entomol..

[52] Delvare G, Lasalle J (1993). A new genus of Tetrastichinae (Hymenoptera: Eulophidae) from the Neotropical region, with the description of a new species parasitic on key pests of oil palm. J. Nat. Hist..

